# Patterned ELR‐Gelatin Hydrogels Enable Rapid Endothelial Monolayer Formation via Bioactive Matrix Chemistry and Surface Topography

**DOI:** 10.1002/adhm.71570

**Published:** 2026-08-10

**Authors:** Jagoda Litowczenko, Yannick Richter, Martyna Michalska, Piotr Paczos, Karine Tadevosyan, Daniel Uribe, Jose Carlos Rodriguez‐Cabello, Ioannis Papakonstantinou, Angel Raya

**Affiliations:** ^1^ Center For Advanced Technologies Adam Mickiewicz University in Poznań, Uniwersytetu Poznańskiego Poznań Poland; ^2^ Stem Cell Potency Group, Regenerative Medicine Program Bellvitge Biomedical Research Institute (IDIBELL) L´Hospitalet De Llobregat Barcelona Spain; ^3^ Program For Clinical Translation of Regenerative Medicine in Catalonia (P‐[CMRC]) L´Hospitalet De Llobregat Barcelona Spain; ^4^ Center For Networked Biomedical Research On Bioengineering Biomaterials and Nanomedicine (CIBER‐BBN) Madrid Spain; ^5^ Department of Chemistry and Chemical Biology TU Dortmund University Dortmund Germany; ^6^ Department of Mechanical Engineering University College London Torrington Place London UK; ^7^ Manufacturing Futures Lab University College London London UK; ^8^ Poznań University of Technology Poznań Poland; ^9^ Institut De Microelectrònica De Barcelona IMB‐CNM (CSIC), Bellaterra Barcelona Spain; ^10^ BIOFORGE Lab, LADIS University of Valladolid, CIBER‐BBN Valladolid Spain; ^11^ Photonic Innovations Lab Department of Electronic & Electrical Engineering University College London London UK; ^12^ Institució Catalana de Recerca i Estudis Avançats (ICREA) Barcelona Spain; ^13^ Physiological Sciences Department Faculty of Medicine and Health Sciences University of Barcelona L´Hospitalet De Llobregat Barcelona Spain

**Keywords:** elastin‐like recombinamers, endothelial monolayer formation, iPSC‐derived endothelial cells, micro/nanopatterned hydrogels, soft lithography

## Abstract

The endothelialization of organ‐on‐chip platforms and vascular implants is often limited by slow cell attachment and unstable monolayer formation. This work presents a scalable workflow that imprints micro‐ and nano‐gratings into elastin‐like recombinamer (ELR)‐based hydrogels, enabling rapid endothelial cell capture and accelerating monolayer formation within 14 days. Three gelatin‐ELR formulations are engineered, with 1H‐NMR confirming incorporation of sequences designed to modulate bioactivity (ELR1: inert, ELR2: uPA‐responsive, ELR3: RGD‐adhesive). ELR incorporation generates fibrillar microstructures and enhances mechanical performance, yielding elastic‐dominant networks suitable for high‐fidelity pattern transfer. Using this library, the combined effects of ELR bioactivity and groove geometry on human iPSC‐derived endothelial cells are evaluated. In a 15‐min attachment assay, patterned ELR composites markedly improve cell retention compared to gelatin, with ELR2 on ∼350 and ∼4 µm grooves performing best, consistent with controlled, cell‐mediated interfacial remodeling. This early advantage persists, as ELR2 and ELR3 hydrogels support rapid alignment and reach confluence by day 14, whereas gelatin remains subconfluent. By combining enhanced early capture with protease‐regulated remodeling, ELR2 identifies a favorable design window. These results establish a materials design framework linking programmable ELR chemistry with surface topography to engineer endothelial interfaces, providing a versatile platform for vascular biomaterials and microphysiological systems.

## Graphical abstract

Micro‐ and nano‐patterned elastin‐like recombinamer (ELR) hydrogels integrate bioactive matrix chemistry with defined surface topography. The combined cues promote rapid endothelial cell capture, alignment, and monolayer formation on soft substrates. This tunable material platform enables robust endothelialization of compliant biomedical interfaces.

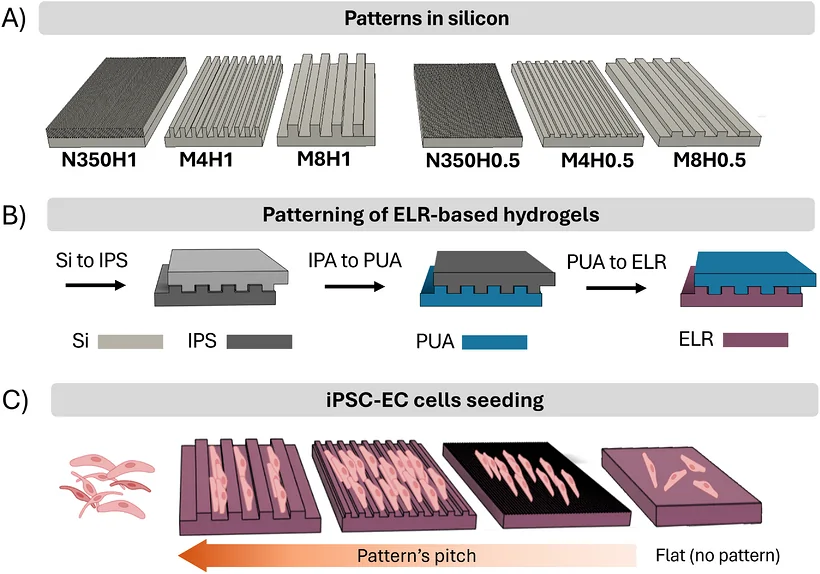

## 1 Introduction

A confluent, quiescent endothelium is essential for controlling thrombosis, inflammation, and barrier function at blood–material interfaces. However, technologies such as organ‐on‐chip platforms and vascular implants (e.g., small‐diameter grafts) are often limited by slow and unstable endothelialization. This is partly because the durable polymers most commonly used compromise endothelial attachment, long‐term patency, and provide neither the biochemical nor topographical cues for stable monolayer formation [1, 2].

Surface topography is a potent physical cue: submicron to micron‐scale parallel grooves can direct protrusion dynamics, focal‐adhesion maturation, and cytoskeletal organization, promoting endothelial elongation, alignment, directed migration, and retention—even under shear [3, 4, 5, 6, 7, 8, 9, 10]. Yet, most parametric studies on topographical guidance have been conducted on rigid materials or PDMS leaving a gap in our understanding of how identical geometries influence cells on soft, protein‐mimetic hydrogels that better emulate the native basement‐membrane mechanics [10, 11].

To address this, we turn to elastin‐like recombinamers (ELRs), which provide a sequence‐defined route to soft matrices with modular bioactivity (e.g., RGD, protease‐responsive motifs), mild processing conditions, optical clarity, and tunable elasticity [12, 13, 14, 15]. As opposed to topographies typically studied on static surfaces, this enables us to investigate them within degradable, cell‐remodeled hydrogels. Concurrently, induced pluripotent stem cell–derived endothelial cells (iPSC‐ECs) offer a renewable human cell source, but their sensitivity to the seeding microenvironment necessitates strategies that enhance rapid capture and accelerate time‐to‐confluence through stable early integrin anchorage [16, 17, 18, 19, 20].

Despite significant progress in hydrogel engineering, no existing platform combines optical clarity, rapid imprintability and compatibility with curved, lumen‐like geometries—three prerequisites for generating prepatterned endothelia capable of withstanding physiological shear stress in organ‐on‐chip systems and small‐diameter vascular grafts. Addressing this unmet technological need requires materials that couple minute‐scale endothelial capture with long‐term monolayer stability while retaining optical clarity, imprintability, and compatibility with lumen‐facing geometries. The novelty of this work is the integration of three elements that are usually studied separately: sequence‐defined ELR bioactivity, soft hydrogel mechanics, and micro/nano‐scale contact‐guidance geometries within one experimentally mapped endothelialization platform.

Here, we design an optically clear, rapidly imprintable gelatin–ELR composite hydrogel. By varying the ELR sequence (ELR1–3: ELR1, a nonbioactive baseline; ELR2, a protease‐responsive variant specifically degradable by uPA; and ELR3, an RGD‐containing adhesive variant), we tune both mechanics and bioactivity relative to a gelatin control, which is inherently cell‐adhesive but lacks defined, tunable adhesion and remodeling cues. We then combine this chemistry with fast soft‐lithographic imprinting to create a library of patterned hydrogels featuring nano‐ (N1/N2) and microscale (M1–M4) gratings of low aspect ratios (<1), alongside flat controls (F). This design allows us to systematically deconvolute the effects of biochemical composition (ELR sequence) and topography (grating spacing and depth) within a single, culture‐compatible system. We selected two representative length scales that correspond to well‐established endothelial sensing regimes. Micron‐scale gratings (4–8 µm width; 0.5–1 µm height) fall within the dimensions repeatedly shown to guide endothelial elongation, junction alignment, and collective migration on rigid and PDMS substrates [4, 8], and include recent demonstrations of distinct guidance mechanisms in single cells versus monolayers on similar microstructured surfaces [6]. In contrast, the ∼350 nm width regime probes the submicron domain associated with integrin clustering, filopodial probing, and nascent adhesion formation, which initiate contact‐guidance responses on nanopatterned substrates [4]. Because most evidence for both scales originates from stiff materials, we used shallow aspect ratios (<1) to ensure reliable replication and optical clarity in soft ELR–gelatin hydrogels. This micro/nano subset therefore provides a biologically meaningful and fabrication‐robust window for isolating geometric effects in compliant protein matrices.

Using this framework, we map how chemistry and geometry regulate: (i) initial adhesion (minutes), (ii) early contact guidance, and (iii) monolayer expansion over 2 weeks, and we established a deployable chemistry‐geometry palette for rapid, stable patterned endothelialization. Finally, by optimizing the viscoelastic properties of the gelatin–ELR blend and thin, flexible intermediate stamps, make this imprinting strategy inherently compatible with replication on gently curved surfaces and within tubular molds, extending this approach to the lumen‐facing geometries relevant to vascular models and devices [3, 4, 5, 6, 7, 8, 9, 10, 12, 13, 14, 16, 17, 18, 19, 21, 22].

Overall, we provide an experimentally grounded map of how ELR content enhances gelatin hydrogels to form rapid, stable endothelial monolayers, advancing strategies for vascular tissue engineering [3, 4, 5, 6, 7, 8, 9, 12, 13, 14, 16, 17, 18, 19], and align with broader evidence that micro/nanopatterns regulate cellular behavior across lineages [21, 22]. Rather than focusing on a single mechanism, this work aims to establish a design framework for endothelial interface engineering, linking matrix bioactivity, mechanics, and surface geometry. Importantly, achieving rapid and stable endothelialization on compliant hydrogel substrates remains a major challenge, as soft matrices often fail to support sufficient cell adhesion and retention. Here, we address this limitation by combining sequence‐defined ELR bioactivity with controlled surface topography within a single tunable material platform, enabling direct comparison of how chemistry, mechanics, and geometry jointly regulate endothelial capture, alignment, and junctional organization from minutes to weeks.

## 2 Materials and Methods

### 2.1 Development of Patterned ELR‐Based Hydrogels

#### 2.1.1 Fabrication of Nano/Micropatterned Silicon Masters

Nanogratings and microgratings were defined on Si wafers by Lloyd's‐mirror laser interference lithography (LIL) and direct laser writing (Heidelberg DWL 66+ laser writer lithography system), respectively. Wafers were solvent‐cleaned, O_2_‐plasma activated (100 W, 60 s, O_2_ 7 sccm), hexamethyldisilazane (HMDS)‐primed, spin‐coated with a photoresist (ma‐N 405 at 300 nm thickness for LIL and S1818 at 1.8 µm for DWL), soft‐baked, then exposed via LIL to achieve 700 nm pitch or via DWL for 8 and 16 µm pitches. After development, patterns were transferred into silicon by Bosch process in inductively coupled plasma‐reactive ion etching (ICP‐RIE) to the target heights H of ∼1.0 and ∼0.5 µm [23].

##### 2.1.1.1 Pattern Encoding and Nomenclature

To ensure consistent terminology across all experiments, we refer to each topographical pattern using a unified abbreviated code in the main manuscript and figures. The full structural parameters and their corresponding codes are: F (Flat), N1 (N350H1), N2 (N350H0.5), M1 (M4H1), M2 (M8H1), M3 (M4H0.5), and M4 (M8H0.5).

Codes denote the groove width (N = 350 nm; M = 4–8 µm) and relief height (H1 = 1 µm; H0.5 = 0.5 µm). The gratings have duty cycle of 0.5, thus grove and ridge widths are the same corresponding to pitch of 700 nm, 8 and 16 µm. Full mapping is provided in Table S1, which includes groove width and height parameters for clarity.

#### 2.1.2 Engineering of ELR‐Based Hydrogels

We engineered imprintable composite hydrogels by blending gelatin with elastin‐like recombinamers selected to decouple basal mechanics from bioactivity. Three ELRs were used throughout: VKV24 (no specific adhesion or protease‐sensitive motifs; referred to hereafter as ELR1), DRIR (contains the slowly urokinase‐type plasminogen activator (uPA)‐degradable DRIR sequence; ELR2), and HRGD6 (presents RGD adhesion motifs; ELR3). Since endothelial cells orchestrate local fibrinolysis and pericellular matrix remodeling through the uPA/uPAR system, which plays a key role in VEGF‐driven angiogenesis and endothelial migration [24], embedding uPA‐degradable sequences within ELRs provides a physiologically relevant mechanism for cell‐mediated remodeling of the coating. This, in turn, is expected to facilitate endothelial spreading, migration, and stable monolayer formation under vascular‐like conditions. These sequences and their biophysical rationale follow prior ELR design frameworks and were produced and characterized as previously reported for VKV‐, DRIR‐ and RGD‐bearing ELRs [25, 26].

##### 2.1.2.1 Stock Solutions

Type A porcine gelatin (Sigma G1890) was dissolved in DMEM, sterile‐filtered (0.22 µm). ELRs were dissolved at 50 mg mL^−^^1^ in PBS (0.22 µm filtered). Phases were combined 0.75 (gelatin/DMEM): 0.25 (ELR/PBS) and cycled on ice/RT/30°C until homogenous and slightly opalescent.

##### 2.1.2.2 Crosslinking

Microbial transglutaminase (mTG) was added immediately before casting (manufacturer's activity; details in Supporting Information), forming ε‐(γ‐glutamyl)‐lysine bonds between gelatin and ELR lysines.

#### 2.1.3 Pattern Transfer Into ELR Hydrogels

To fabricate patterned ELR hydrogels, three lithographic steps were followed: (iii‐i) transfer from masters to intermediate polymer stamps (IPS) via nanoimprint lithography (NIL) to preserve the master longevity, (iii‐ii) transfer from IPS to poly(urethane acrylate) (PUA) for scalable replication of the patterning workflow, and (iii‐iii) transfer from PUA to ELR composite by stamping.

##### 2.1.3.1 NIL (Master → IPS)

Briefly, prior to NIL, masters were silanized in trichlorooctadecylsilane for 60 min (1% v/v toluene solution) and postannealed (120°C, 30 min) to facilitate demolding [27]. Pattern dimensions were verified by scanning electron microscopy (SEM, Zeiss) and optical profilometry (Keyence VHX‐series microscope) before replication. The NIL process was performed on EITRE 3 (Obducat). The IPS (Obducat) stamping process was operated at 145°C and 30 bar for 20 s.

##### 2.1.3.2 Soft‐Lithographic Casting of PUA Daughter Stamps (IPS → PUA)

PUA resin was compounded according to our previous protocols (ACSNano 2020,14,12091−12100). Briefly, diacrylate prepolymer (Ebecryl 284, Allnex) was blended with 30 wt% trimethylolpropane‐ethoxylate triacrylate (Sigma 409073) as reactive diluent; photoinitiators Irgacure 184 (BASF 30472119) and 2‐hydroxy‐2‐methylpropiophenone (Sigma 405655) were each added at 1.5 wt% relative to total resin; TEGO Rad 2200N (1 wt%) improved wetting/release. The mixture was vortexed, degassed and stored cold, protected from light. A togetherilm was drop‐cast on IPS, overlaid with a clean glass support to define a uniform, thin stamp, and UV‐cured for ∼20 s at 365 nm (high intensity), followed by a 10 h low‐intensity postcure. Stamps were gently demolded to yield flexible PUA negatives suitable for repeated use. Before cell work, stamps were rinsed in isopropanol, air‐dried, sterilized in 70% ethanol and UV‐irradiated.

##### 2.1.3.3 Soft‐Lithographic Stamping of ELR‐Based Hydrogels (PUA → ELR Hydrogel)

Freshly prepared ELR–gelatin (with mTG) blends were dispensed into 48‐well tissue‐culture plates at 0.50 mL per well using a positive‐displacement pipette to ensure volumetric accuracy and minimize bubble entrainment. After casting, plates were left at room temperature of 21°C (RT) for 3 min to allow incipient gelation (pre‐set), providing sufficient viscoelasticity for imprinting without loss of conformity.

For topography transfer, a thin PUA stamp carrying the line pattern was gently placed pattern‐side down onto each preset hydrogel surface, ensuring full areal contact and avoiding lateral sliding. The hydrogel–stamp assemblies were maintained at RT for 10 min, then transferred to 4°C for a further 10 min to stabilize the imprinted interface while the mTG reaction progressed. Plates were subsequently removed to RT and kept undisturbed for an additional ∼40 min to allow completion of crosslinking. Stamps were peeled, gels rinsed in PBS and equilibrated in medium prior to seeding. All steps were performed under sterile conditions; stamps were handled with sterile forceps and lowered vertically to avoid trapping air. Minimal pressure was applied only to ensure conformal contact; no external load was used.

### 2.2 Hydrogel Characterization

#### 2.2.1 Oscillatory Rheology

Small‐amplitude oscillatory rheology was performed on a stress‐controlled rheometer (Anton Paar MCR‐302) using a 25 mm serrated parallel‐plate geometry with a gap of 1.0 mm. Samples were loaded at 25°C, trimmed, and equilibrated for 5 min. Strain‐sweep tests (0.015–100%) at 1 rad s^−^^1^ identified the linear viscoelastic regime (LVR). Frequency sweeps (0.1–100 rad s^−^^1^) were then run at *γ* = 0.5% (within LVR). Temperature was controlled with a Peltier stage (±0.1°C). Storage (G′), loss (G″) moduli, and complex viscosity (η*) were exported as mean ± SD across *n* = 3 hydrogels per formulation.

#### 2.2.2 Uniaxial Tensile Testing

Dog‐bone‐like strips (width 5 mm, gauge length 15 mm, thickness 1.5–2 mm) were cut from fully cross‐linked gels and tested in tension on a Zwick/Roell Z100 with a 5 kN load cell (class 1 accuracy to 0.2% Fnom). Custom flat grips used 61 HRC steel inserts coated with 95 ShA polyurethane to prevent slippage/necking of soft gels. Tests were run at 60 mm min^−^^1^ from a 0.1 N preload to failure. True stress–strain curves were computed by engineering‐to‐true conversion assuming incompressibility (Poisson ∼0.5) within the large‐strain regime. We report ultimate tensile strength (UTS), strain at break, Young's modulus (tangent at 5%–10% strain), and stiffness (N mm^−^^1^); *n* = 4 per material.

#### 2.2.3 ^1^H NMR of Composites

Proton spectra (^1^H NMR) of ELR components and Gelatin‐ELR composites were acquired on a Bruker AVANCE III 400 MHz at 298 K using standard single‐pulse acquisition with water presaturation. Chemical shifts are referenced to TSP (0.00 ppm) in D_2_O. Spectra were phase‐ and baseline‐corrected in MestReNova, then overlaid after intensity normalization. Representative fingerprints differentiating VKV24 (ELR1) and HRGD6 (ELR3) are shown in Figure 2L. Representative ^1^H‐NMR fingerprints for all gelatin‐ELR composites are shown in Figure 2K,L, including the diagnostic valine and isoleucine‐associated regions used to confirm ELR incorporation and distinguish the formulations. Full peak lists are in Supporting Information.

### 2.3 Human iPSC Culture

All work with human iPSC lines was conducted under approval from the Spanish competent authorities (Commission on Guarantees concerning the Donation and Use of Human Tissues and Cells, Instituto de Salud Carlos III). Ethical Statement: This study was performed in accordance with the Declaration of Helsinki. The use of human induced pluripotent stem cells (hiPSCs) was approved by the Commission on Guarantees concerning the Donation and Use of Human Tissues and Cells of the Carlos III Spanish National Institute of Health, Spain, under approval number 07/2023. The FiPS Ctrl1‐mR5F‐6 line (registered at the Spanish National Stem Cell Bank) was maintained on Matrigel‐coated cultureware (Corning) in mTeSR1 medium (STEMCELL Technologies). Medium was renewed daily except on the first day after passaging. For routine expansion, colonies were split 1:4–1:6 by brief incubation with 0.5 mM EDTA (Invitrogen) at 37°C (∼2 min) and re‐plated as small aggregates onto fresh Matrigel for continued maintenance.

### 2.4 Differentiation of Endothelial Cells

Endothelial differentiation was adapted from established lateral‐mesoderm protocols [16] hiPSCs on Matrigel were dissociated to single cells with Accutase (37°C, ∼8 min) and seeded onto Matrigel‐coated 12‐well plates at 1.5 × 10^5^ cells/well in mTeSR1 with 10 µM Y‐27632 (ROCK inhibitor, Sigma). After 24 h, medium was switched to EC priming medium (N2B27 composed of DMEM/F‐12:Neurobasal 1:1 with N2 and B27 supplements) containing 8 µM CHIR99021 (GSK3β inhibitor; Sigma, SML1046) and 25 ng mL^−^^1^ BMP4 (R&D Systems) for 3 days. Cultures were then transferred to EC induction medium (StemPro‐34 SFM; Thermo Fisher) supplemented with 200 ng mL^−^^1^ VEGF (PeproTech) and 2 µM forskolin (Sigma‐Aldrich) for a further 3 days.

### 2.5 Expansion and Purification of Endothelial Cells

On day 6, cells were re‐plated onto 0.1% gelatin‐coated dishes and expanded in EGM‐2 (Lonza) supplemented with a YAC cocktail (3 µM CHIR99021, 10 µM Y‐27632, and 10 µM SB431542) for ∼4 days. Purification was performed by sequential differential detachment with TrypLE (Thermo Fisher): after two PBS rinses, cultures received TrypLE at room temperature until non‐EC contaminants detached with gentle tapping and were removed; remaining ECs were then exposed to TrypLE at 37°C (∼5 min), collected, and re‐plated on 0.1% gelatin. iPSC‐ECs at passages 2–3 were used for all experiments.

### 2.6 Immunostaining Analysis

Hydrogel constructs (or control coverslips) were fixed in 4% paraformaldehyde (PFA; Sigma) for 15–30 min (RT or 4°C), rinsed in TBS (3 × 10–30 min) and permeabilized/blocked in TBS with 0.1% Triton X‐100 and 6% donkey serum (Chemicon). Primary antibodies were diluted in blocking buffer and incubated at 4°C with gentle agitation (overnight to 72 h depending on target). After 4 TBS washes, fluorescent secondary antibodies were applied for 2 h at RT (light‐protected) or overnight at 4°C, followed by DAPI nuclear counterstaining. F‐actin was labeled with fluorescent phalloidin. Imaging was performed on a Zeiss LSM980 Airyscan2; image processing used FIJI/ImageJ and, where indicated, Imaris (Microscopy Image Analysis Software, Oxford Instruments). Primary and secondary antibodies used are listed in Table S2.

### 2.7 Quantitative Real‐Time Polymerase Chain Reaction (qRT‐PCR)

Total RNA was extracted from iPSC‐ECs using Maxwell RSC simplyRNA Cells kits on a Maxwell RSC instrument (Promega). cDNA was synthesized from 1 µg RNA using Transcriptor First Strand cDNA kit (Roche). qPCR was run on a LightCycler 480 (Roche) with GAPDH as housekeeping control. RNA from primary HUVECs served as a positive endothelial control, and human dermal fibroblast RNA as a negative control. Primer sequences are listed in Table S2.

### 2.8 Live/Dead Assay

Live/dead working solution was prepared in PBS by mixing 50 µL propidium iodide (PI; 2 mg mL^−^^1^ stock in PBS; Sigma‐Aldrich) and 8 µL fluorescein diacetate (FDA; 5 mg mL^−^^1^ stock in acetone; Sigma‐Aldrich). Constructs were rinsed with PBS, incubated with the staining mixture for up to 5 min at RT in the dark, washed with PBS, and imaged immediately in phenol red free DMEM by confocal microscopy.

### 2.9 Hydrogels Topography, Cell Morphology, and Spreading Analysis

Bright‐field images (attachment and coverage assays) were acquired on a digital microscope (Keyence VHX‐series) using identical exposure settings across conditions. iPSC‐EC surface coverage (% area) was quantified in FIJI using background subtraction, auto‐thresholding (Otsu), and binary area fraction within a fixed region of interest; four nonoverlapping fields per condition were analyzed and averaged per well/biological replicate. Orientation was assessed from F‐actin or phase‐contrast images using OrientationJ, reporting angle histograms (0°–180°) and rose plots; alignment indices were computed as described in Methods (binning and smoothing parameters as in Supporting Information). Acquisition parameters (objective, pixel size, z‐sampling) were held constant within each experiment.

## 3 15‐min Attachment Assay (Retrieved‐Cell Quantification)

iPSC‐ECs were seeded at 150 000 cells/cm^2^ in 48‐well plates containing equilibrated hydrogels. After exactly 15 min, wells were gently rinsed once with 200 µL PBS to collect nonadherent (retrieved) cells. The PBS containing detached cells was transferred into microcentrifuge tubes, pelleted at 300 × g for 5 min, resuspended in 200 µL of medium, and counted using an automated cell counter. Retrieved‐cell number was used as an inverse metric of adhesion strength (fewer retrieved cells = stronger immediate attachment). This assay quantifies early adhesion only.

### 3.1 Quantification of Cell Orientation (OrientationJ → Polar Plots)

Based on phase‐contrast or F‐actin images, local orientation was computed in Fiji using the OrientationJ plugin (structure tensor). Angle histograms (0°–180°) were exported and normalized to the sum of bin counts. For visualization, Python/Matplotlib generated polar bar charts with the zero‐angle at the top and the axis displayed from −90° to +90°. Plot opacity/border width were standardized across conditions.

### 3.2 Image Processing and Area‐Coverage Calculation

Confocal/bright‐field images were analyzed by an automated Python pipeline: 1. Preprocessing: grayscale import. 2. Adaptive thresholding: Gaussian method (blockSize = 11, C = 2) to segment cells under non‐uniform illumination. 3. Morphology: closing then opening with a 5 × 5 rectangular kernel to fill intrusions and remove speckle. 5. Contour detection & hole filling: external contours were detected (cv2.RETR_EXTERNAL) and filled (thickness = FILLED). 6. Metric: area covered (%) = 100 × (cell‐pixels)/(total pixels), where cell‐pixels are nonzero entries of the filled mask. 7. Reporting: side‐by‐side panels (original/thresholded/filled) and per‐image coverage were compiled into a multipage PDF; a tab‐delimited file (image name, % coverage) was saved for statistics. 8. Implementation used OpenCV, NumPy and Matplotlib; parameters were held constant within an experiment.

Area coverage (%) was analyzed per day by two‐way ANOVA with factors Material (Gelatin, ELR1–3) and Pattern (F, N1/N2, M1–M4), including the interaction. Assumptions were evaluated on model residuals (Shapiro–Wilk; Brown–Forsythe). When needed, data were logit‐transformed. Posthoc contrasts used Šídák correction for (i) within‐material comparisons (each pattern vs. Flat) and (ii) within‐pattern comparisons (each ELR vs. Gelatin). As complementary kinetics metrics, we computed AUC(0–14 d) for %coverage and the time to 90%–95% coverage (T90/T95) by linear interpolation between timepoints.

#### 3.2.1 Orientation Analysis (OrientationJ) and Area Coverage (Python)

OrientationJ (Fiji, structure tensor), normalization to the sum of bins, polar plots in Matplotlib with 0° at top and axis −90° to +90°. Coverage pipeline: grayscale import → adaptive Gaussian thresholding (blockSize = 11, C = 2) → morphological close/open (5 × 5 kernel) → external‐contour fill → % area = non‐zero / total × 100; panels and TSV exported; code uses OpenCV, NumPy, Matplotlib.

### 3.3 Statistical Analysis

Unless specified, data are mean ± s.d.; technical fields per well were averaged before inference. For 15‐min retrievals (Figure 4) and for coverage (Figure 6), we applied two complementary approaches:

#### 3.3.1 Primary (Per‐Day) Analysis

Two‐way ANOVA at each time point with factors Material (Gelatin, ELR1, ELR2, and ELR3) and Pattern (F, N1, N2, and M1–M4), Type III SS; residual diagnostics (Shapiro–Wilk; Brown–Forsythe); log_10_ transform if needed. Post‐hoc: Šídák for simple effects (pattern vs. flat within material; ELR vs. gelatin within pattern).

#### 3.3.2 Global Analysis (Report in SI)

Linear mixed‐effects model with fixed effects Material, Pattern, Day (3/7/14) and all interactions; random intercept for replicate/well (fields nested), Satterthwaite df; post‐hoc Tukey/Šídák. Provide partial *η* ^2^ (ANOVA) or conditional *R* ^2^ (LMM) as effect sizes and adjusted CIs.

All tests in GraphPad Prism 10 or R (lme4/emmeans); *α* = 0.05.

## 4 Results

### 4.1 A Scalable Workflow for Producing Patterned ELR Hydrogels

**FIGURE 1:**
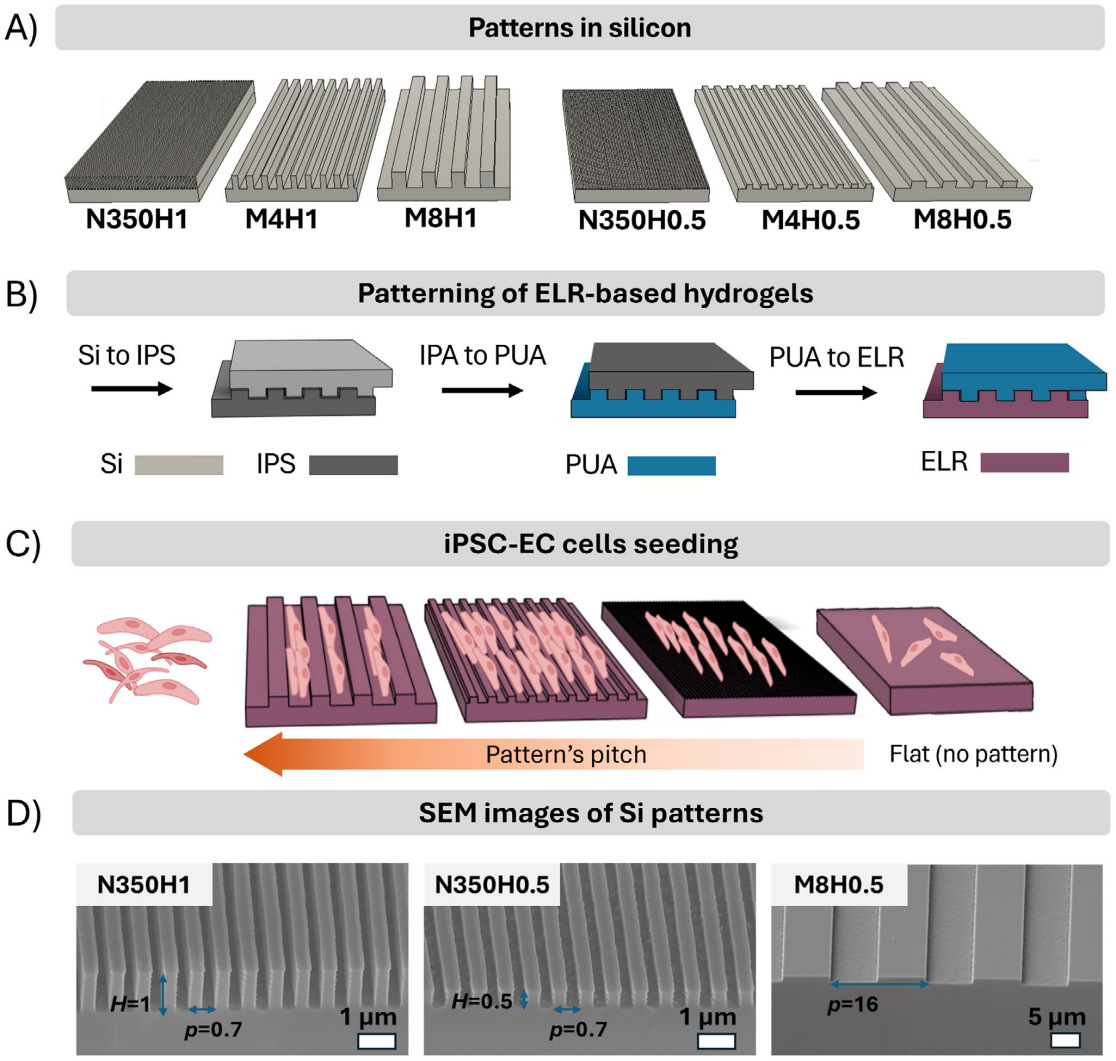
Overview of the fabrication pipeline and experimental workflow. (A) Patterns in silicon: N350H1 (N1), M4H1 (M1), M8H1 (M2), N350H0.5 (N2), M4H0.5 (M3), and M8H0.5 (M4), corresponding to 350 nm, 4, and 8 µm gratings with heights of ∼0.5 or 1 µm, fabricated by LIL/DWL and ICP‐RIE. (B) Three‐step pattern transfer workflow: Si → IPS (NIL), IPS → PUA, and PUA → ELR hydrogel. (C) Representative patterned ELR hydrogels after imprinting and demolding and seeding of iPSC‐derived endothelial cells (iPSC‐ECs) onto patterned hydrogels, followed by readouts for immediate attachment, alignment, and coverage. (D) SEM micrographs of silicon masters (N350H1, N350H0.5, and M8H0.5). Scale bars as indicated.

We established a scalable pipeline to generate line‐patterned gelatin‐ELR hydrogels. Using soft lithography, we replicated a library of micro‐ and nano‐gratings from silicon masters onto intermediate polymer stamps (IPS), and finally into polyurethane acrylate (PUA) daughter stamps for reliable imprinting (Figure 1). This method faithfully transferred topographies across centimeter‐scale areas, creating a library of patterns from nanometer (N1/N2) to micrometer (M1–M4) scales, alongside flat controls (F), suitable for high‐resolution live‐cell imaging.

Profilometry confirmed excellent pattern fidelity for micron‐scale gratings, while nanoscale grooves (350 nm) showed reduced feature height, indicating partial amplitude loss during imprinting into the viscoelastic hydrogel. Pattern replication was robust across all material formulations (Figures S1–S6).

### 4.2 ELR Composites Enhance Mechanical and Structural Properties

**FIGURE 2:**
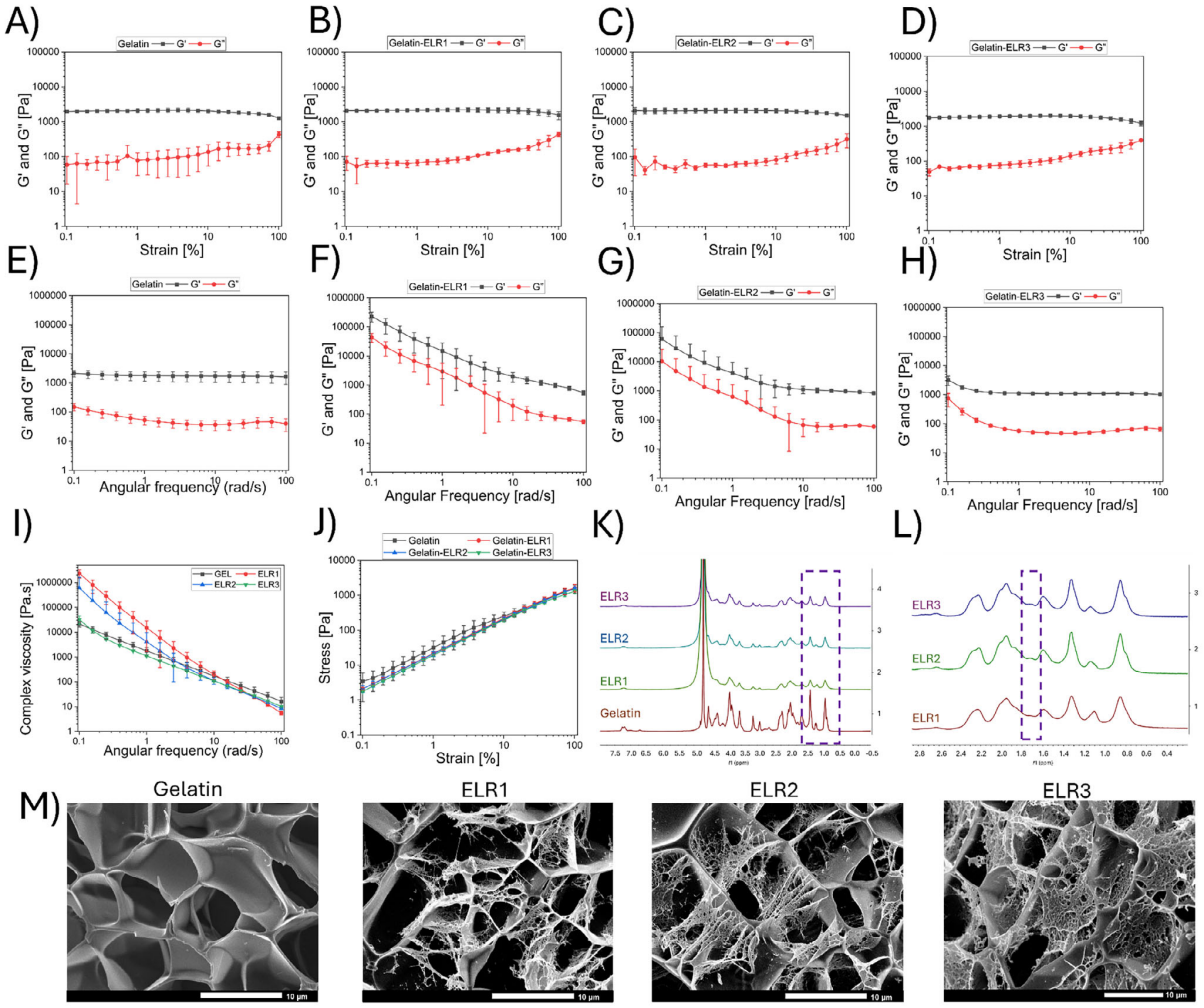
Mechanical and molecular characterization of gelatin–ELR hydrogels. (A–D) Strain (amplitude) sweeps showing storage (G′) and loss (G″) moduli as a function of strain, demonstrating predominantly solid‐like viscoelastic behavior (G′ > G″) across all formulations. (E–H) Frequency sweeps showing storage (G′) and loss (G″) moduli as a function of angular frequency. (I) Complex viscosity (η*) as a function of angular frequency. (J) Stress response as a function of strain.(K–L) ^1^H‐NMR overlays of gelatin and gelatin–ELR composites. Panel K highlights the ∼0.9 ppm region, where increased intensity in ELR1, ELR2, and ELR3 reflects valine isopropyl protons, confirming ELR incorporation. Panel L shows a zoom of the ∼1.7 ppm region, where ELR2 and ELR3 exhibit a more pronounced shoulder attributed to isoleucine methylene protons, consistent with their higher Ile content compared with ELR1 (VKVx24). These resonances collectively verify blending and allow molecular differentiation between ELR variants. (M) Cryo‐SEM micrographs revealing a porous, fibrillar architecture in all hydrogels. Scale bar, 10 µm.

We synthesized three gelatin‐ELR hydrogels via enzymatic crosslinking with microbial transglutaminase, incorporating ELRs with distinct functionalities: VKV24 (ELR1, bioinert baseline), DRIR (ELR2, uPA protease responsive), and HRGD6 (ELR3, RGD‐adhesive). ^1^H‐NMR confirmed successful ELR incorporation, indicated by increased signal at ∼0.9 ppm (valine isopropyl methyl groups) and distinct signatures of ELR2/3 at ∼1.7 ppm (isoleucine side chains) (Figure 2K,L).

Rheological characterization within the linear viscoelastic regime (*γ* = 1%) confirmed stable, gel‐like behavior (G′ > G″) for all formulations (Figure 2A–J). ELR incorporation enhanced the matrix resistance to deformation, with ELR2 and ELR3 composites sustaining higher stresses at matched strains than gelatin or ELR1 (Figure 2J). Frequency sweeps and complex viscosity profiles were consistent with robust, shear‐thinning viscoelastic networks (Figure 2I).

Tensile testing revealed that ELR composites exhibited elastomeric behavior with high extensibility before rupture (Figure S7). ELR3 achieved the highest ultimate tensile strength, albeit with wide variability, while ELR2 provided the most consistent and highest Young's modulus, offering a favorable balance of stiffness and reproducibility (Figure S7). Cryo‐SEM imaging revealed that ELR incorporation transformed the gelatin matrix into a more open, fibrous, and interconnected network, with ELR2 and ELR3 displaying finer fibrillar meshes consistent with their superior mechanical performance (Figure 2M). Collectively, these data show that incorporating ELRs stabilizes the gelatin matrix against large‐amplitude damage while preserving culture‐relevant, elastic‐dominant mechanics (Figure 2A–J) [2, 10, 11, 15, 26].

### 4.3 Generation and Characterization of iPSC‐Derived Endothelial Cells

**FIGURE 3:**
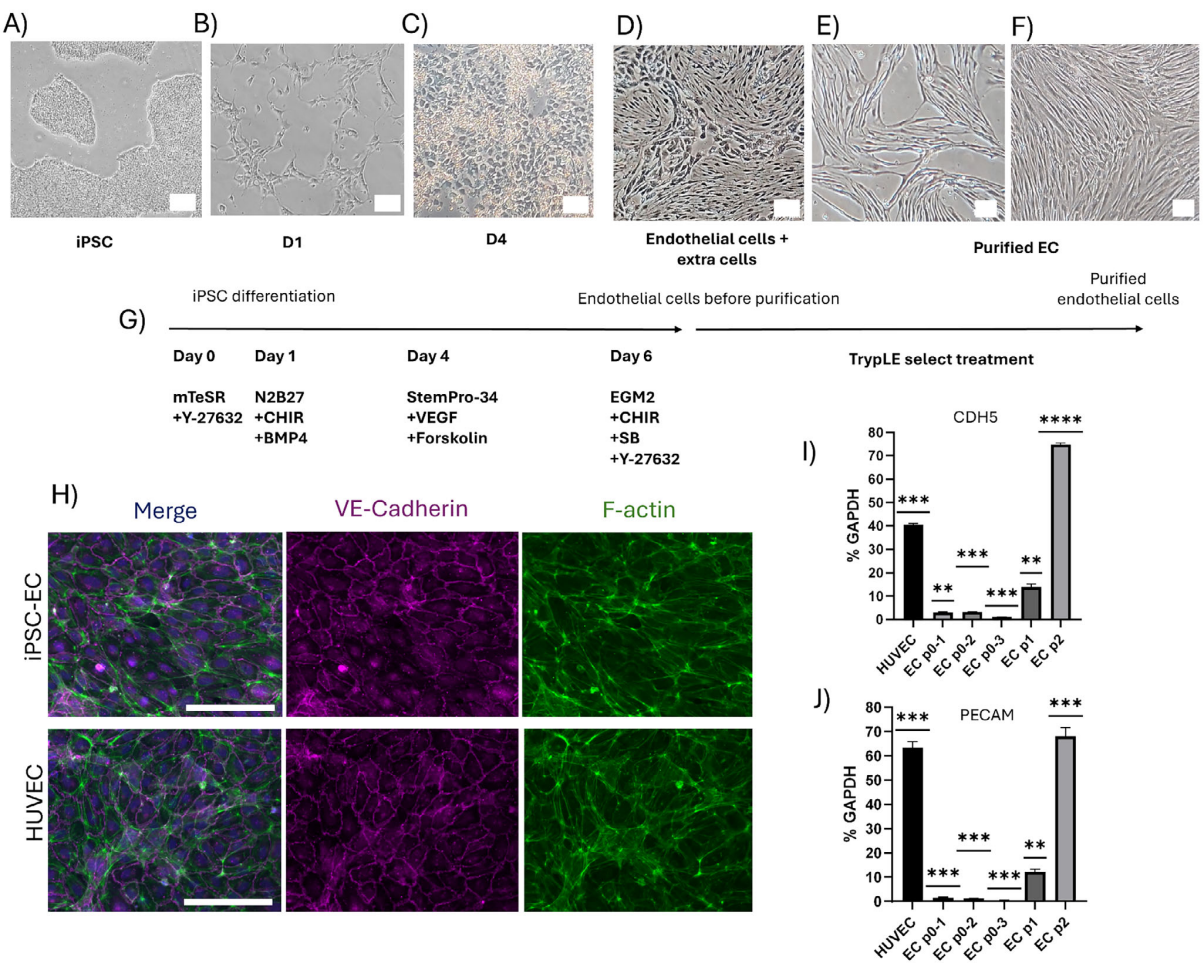
Generation and characterization of human iPSC‐derived endothelial cells (iPSC‐ECs). (A–D) Bright‐field images illustrating morphology at days 0, 1, 4, and 6 of differentiation. Scale bar, 100 µm. (E) Endothelial cells before purification. (F) Purified endothelial cells after expansion. (G) Schematic of the endothelial differentiation and purification workflow. (H) High‐magnification dual immunofluorescence staining showing VE‐cadherin‐positive cell‐cell junctions and F‐actin organization in iPSC‐ECs and HUVEC positive controls. VE‐cadherin, magenta; F‐actin, green; nuclei, DAPI/blue. (I) qRT‐PCR analysis of endothelial marker CDH5 expression across passages 1–2; HUVEC serves as a positive control. Data are Mean ± SD (*n* = 3). (J) qRT‐PCR analysis of endothelial marker PECAM1 expression across passages 1–2; HUVEC serves as a positive control. Data are Mean ± SD (*n* = 3).

We differentiated iPSC into endothelial cells (iPSC‐ECs) using a Wnt/BMP‐guided protocol [16] (Figure 3A–G). The resulting cells exhibited cobblestone endothelial morphology and formed continuous VE‐cadherin‐positive junctions with organized F‐actin structures, comparable to HUVEC controls (Figure 3H and Figure S12). qRT‐PCR confirmed expression of canonical endothelial markers (Figure 3I,J): a junctional cadherin (CDH5, PECAM1/KDR) at levels comparable to HUVECs, confirming a stable endothelial phenotype suitable for downstream assays [16].

### 4.4 Combined Effects of Matrix Chemistry and Surface Topography on Immediate Cell Attachment

**FIGURE 4:**
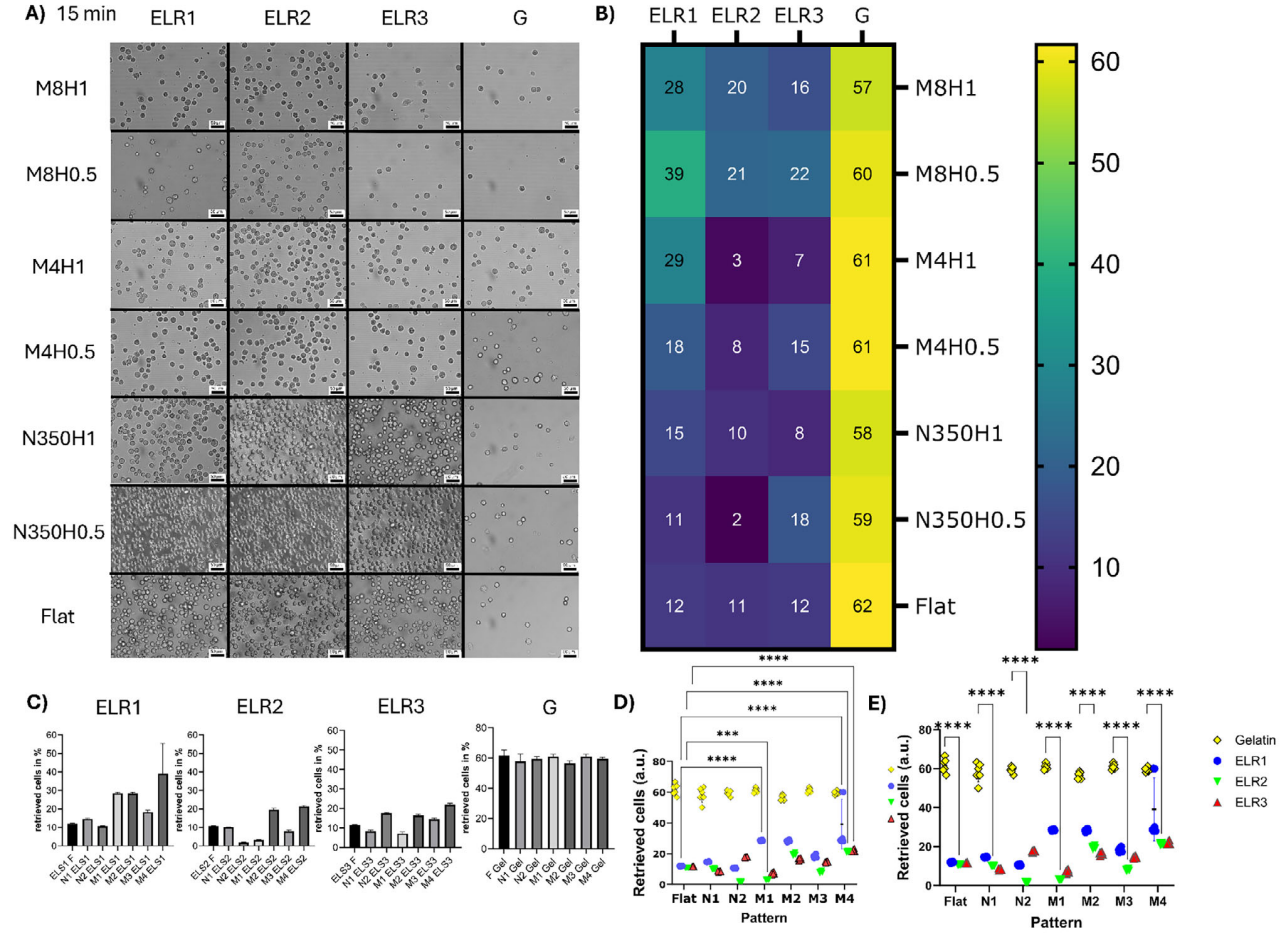
Impact of topography and ELR formulation on immediate iPSC‐EC attachment (15 min). (A) Representative bright‐field micrographs 15 min after seeding, after removal of nonadherent cells by gentle washing. Rows show full pattern identifiers (M8H1, M8H0.5, M4H1, M4H0.5, N350H1, N350H0.5, and Flat), columns show coating materials (ELR1–3, Gelatin). (B) Heat map of cells retrieved from the supernatant (lower values = stronger immediate attachment). Tile numbers indicate mean values (a.u.). (C) Quantification of retrieved cells for each coating–pattern combination (mean ± SD; *n* = 6). (D) Comparison of retrieved cells across abbreviated pattern groups (F, N1–N2, and M1–M4). (E) Comparison across coating materials (Gelatin, ELR1–3). Statistical analysis: two‐way ANOVA with Šídák's multiple‐comparisons test (* *p* < 0.05, ** *p* < 0.01, *** *p* < 0.001, **** *p* < 0.0001). Note: Panels A–C display full pattern names; panels D–E use abbreviated codes (F, N1–N2, and M1–M4) for readability. Mapping between full names and abbreviations is provided in Table S1.

A critical 15‐min attachment assay revealed that both hydrogel chemistry and surface topography profoundly influenced initial cell adhesion. ELR‐based surfaces retained significantly more cells than gelatin across all patterns (Figure 4A–E). Quantitative analysis showed a several‐fold reduction in retrieved (nonattached) cells on ELR substrates compared to gelatin (Figure 4B). Patterns significantly modulated early cell retention. Among all geometries, the micron‐scale M1–M2 grooves produced the lowest number of retrieved cells, indicating the strongest immediate attachment, followed by the sub‐micron N1–N2 patterns, whereas Flat controls consistently showed weaker retention.

Across materials, ELR2 yielded the lowest retrieved‐cell counts for all patterns, consistent with its uPA‐responsiveness and enhanced pericellular remodeling capability. Importantly, comparison of flat conditions shows that all ELR formulations already enhance initial attachment relative to gelatin (Figure 4B), establishing a chemistry‐driven baseline. The further reduction in retrieved cells observed upon patterning—most pronounced for ELR2 on M1–M2 and N2 geometries—indicates that topographical guidance provides an additional, geometry‐dependent contribution to early adhesion.

This chemistry‐driven baseline was further modulated by topography. Specifically, the submicron N2 and micron‐scale M1 grooves further enhanced attachment in combination with ELR chemistry. ELR2 (DRIR) performed exceptionally well on these patterns, yielding the lowest cell retrieval (Figure 4B,C). Two‐way ANOVA confirmed significant independent and interactive effects of both material and pattern (Figure 4D). These results indicate that a protease‐responsive, slowly remodeled interface (ELR2) particularly when combined with selected groove regimes, provides favorable conditions for nascent adhesion formation. The magnitude and speed of this effect are particularly relevant for perfused systems, where the first minutes after seeding determine long‐term lumen coverage. Such rapid attachment under minimal shear‐like washing steps indicates that these chemistries and groove regimes could markedly reduce cell loss during initial perfusion in chips and tubular grafts. The 15‐min attachment assay was intentionally designed to evaluate very early endothelial cell‐material interactions prior to extensive spreading, focal adhesion maturation, proliferation, or matrix remodeling. This short‐term timepoint enables sensitive discrimination of material‐driven endothelial capture and initial adhesion kinetics between different hydrogel formulations and surface topographies. Longer incubation periods would likely reduce the relative differences observed between groups, as progressive spreading and adaptation over time partially mask early attachment‐dependent effects. Notably, rapid endothelial cell attachment observed within 15 min suggests efficient initial cell capture, which is particularly relevant for applications such as vascular grafts where early cell retention is critical. Together, these observations support a model in which ELR chemistry defines the baseline adhesion capacity, while surface topography modulates the magnitude and spatial organization of early cell attachment.

### 4.5 Rapid Contact Guidance and Accelerated Monolayer Formation

**FIGURE 5:**
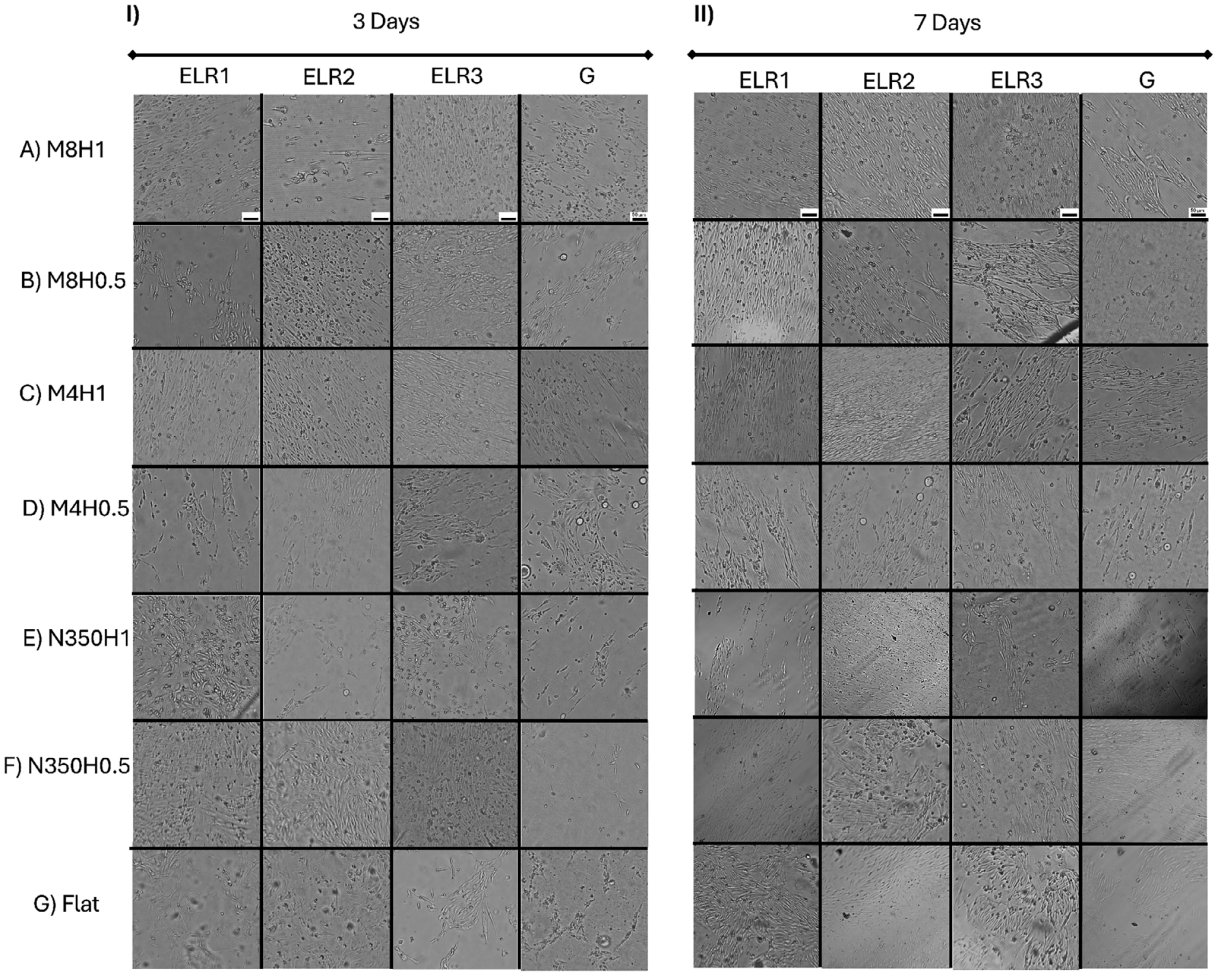
Topography drives early orientation of iPSC‐ECs on ELR hydrogels. (I) Representative phase‐contrast images of iPSC‐EC morphology and local alignment on patterned ELR hydrogels after 3 days of culture. (II) Representative phase‐contrast images of iPSC‐EC morphology and local alignment on patterned ELR hydrogels after 7 days of culture. Scale bars as indicated. Pattern key: F (Flat), N1 = N350H1, N2 = N350H0.5, M1 = M4H1, M2 = M8H1, M3 = M4H0.5, and M4 = M8H0.5.

Within hours of seeding, iPSC‐ECs on patterned ELR hydrogels exhibited pronounced contact guidance. Submicron and micron groove patterns induced rapid axis alignment of iPSC‐ECs, most prominently on M1–M2, followed by N1–N2, whereas Flat substrates produced isotropic spreading. Cells on micron‐scale grooves (M1–M4) showed strong elongation and alignment parallel to the groove axis, while submicron patterns (N1/N2) induced a weaker, broader alignment. Flat controls were isotropic (Figure 5A).

**FIGURE 6:**
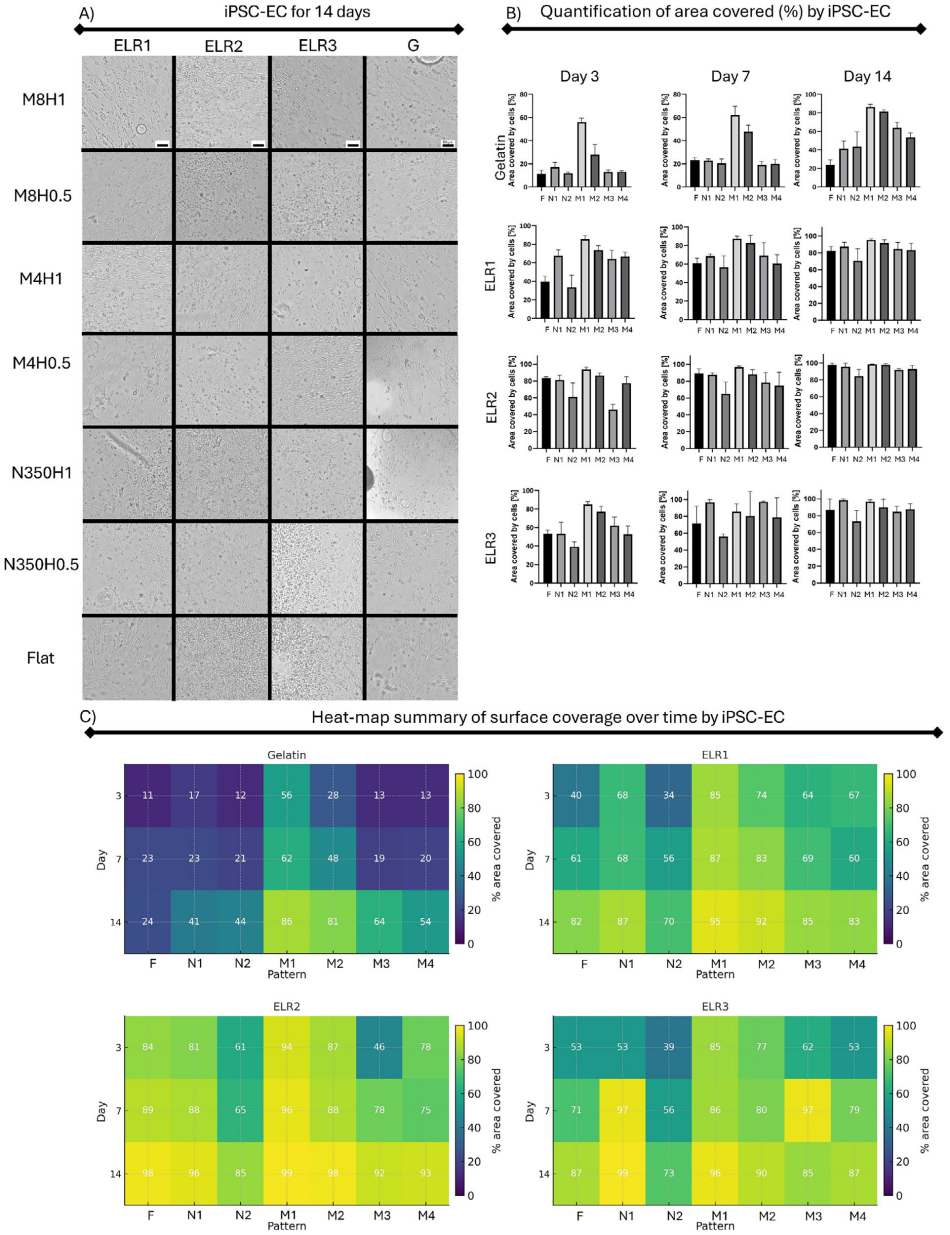
Patterned ELR‐based hydrogels accelerate expansion to confluence relative to gelatin. (A) Representative bright‐field mosaics at day 14 for Gelatin versus ELR1–3 across F, N1/N2, and M1–M4. (B) Quantitative analysis of surface coverage (% area) at days 3, 7, and 14 (bars = mean ± SD; points = replicates). (C) Heat‐map summary of surface coverage over time (Day × Pattern per material; Gelatin, ELR1–3); numbers indicate mean % coverage. Micron grooves (M1–M2) consistently promote faster expansion, with ELR2 achieving the shortest time to near‐confluence. Statistics: Two‐way ANOVA per day (Material × Pattern) with Šídák correction for (a) each pattern versus Flat within a material and (b) each ELR versus Gelatin within a pattern (see legend and Supporting Information).

**FIGURE 7:**
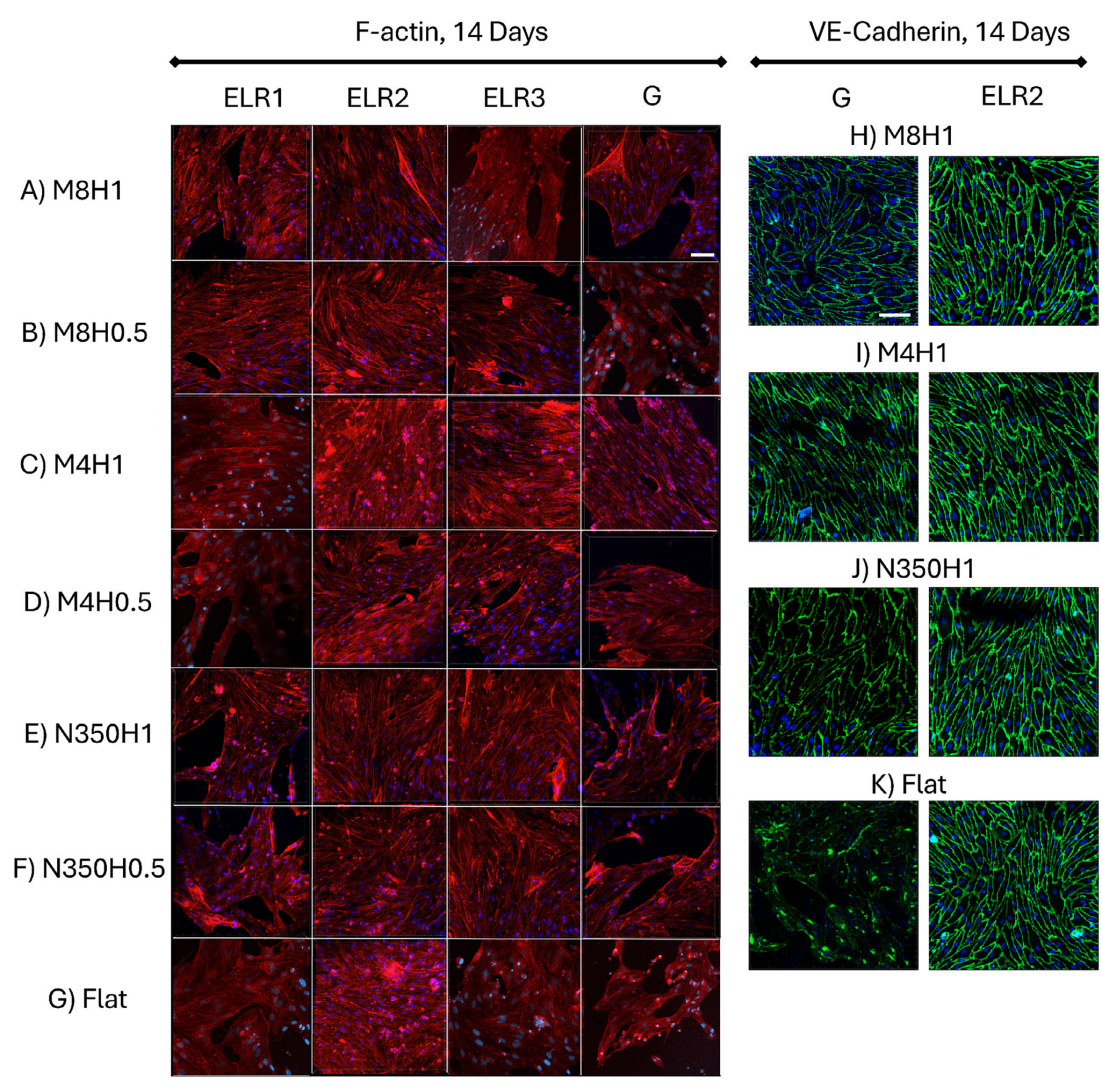
Cytoskeletal organization and endothelial junction formation on patterned ELR‐based hydrogels after 14 days of culture. (A–G) F‐actin staining (phalloidin, red) with DAPI nuclear counterstaining (blue) demonstrates cytoskeletal organization, cell elongation, and contact guidance on M8H1, M8H0.5, M4H1, M4H0.5, N350H1, N350H0.5, and flat substrates. (H–K) Representative VE‐cadherin staining (green) with DAPI (blue) on selected patterned substrates, M8H1, M4H1, N350H1, and flat controls, comparing gelatin and ELR2 conditions. These panels show endothelial junction organization and monolayer continuity in response to substrate topography. ELR‐based patterned hydrogels promoted aligned actin organization and improved endothelial junction continuity compared with gelatin controls, with the strongest alignment observed on micron‐scale groove geometries. Scale bar, 100 µm.

This early guidance translated into accelerated surface colonization. Micron‐scale patterns (M1–M2) accelerated surface coverage at days 3–14 across all ELR hydrogels, with ELR2 consistently reaching the highest coverage and shortest time‐to‐confluence across patterns. By day 3, ELR‐based hydrogels, particularly ELR2, supported significantly greater cell coverage than gelatin (Figure 5A and Figure 6B). This divergence widened over time. By day 7, grooved ELR substrates (especially M1‐M2) approached confluence, while gelatin lagged significantly (Figure 5B and Figure 6B). By day 14, ELR hydrogels were near‐confluent across most patterns, whereas gelatin remained sub‐confluent on all but the most potent topographies (M1–M2) (Figure 6A,B). Analysis of cumulative coverage (AUC) and time‐to‐confluence (T90/T95) confirmed that the combination of ELR chemistry (particularly ELR2) and the 1 µm‐high micron‐scale topographies (M1–M2) yielded the most rapid and complete monolayer formation (Figure 6C and Figure 7).

### 4.6 Cytoskeletal Organization in Confluent Monolayers

After 14 days, F‐actin staining revealed nearly continuous monolayers with well‐developed stress fibers on ELR hydrogels. On micron‐scale grooves (M1–M2) the cytoskeleton was highly organized, with dense, parallel actin stress fibers aligned to the groove axis. Flat ELR substrates yielded confluent cell monolayers with largely isotropic actin organization. Additional VE‐cadherin staining confirmed improved endothelial junction continuity and more organized monolayer architecture on patterned ELR2 hydrogels compared with gelatin controls, particularly on micron‐scale groove geometries (Figure 7H–K). In contrast, cells on gelatin were sparse, poorly spread, and exhibited disordered actin networks (Figure 7). These structural observations corroborate the quantitative area‐coverage and orientation data (Figures 6C and S8–S11), confirming that patterned ELR‐based hydrogels direct the formation of a stable, aligned endothelium.

## 5 Discussion

This study shows that matrix chemistry and surface topography jointly regulate endothelial cell behavior, influencing outcomes from initial attachment to final confluence. We have demonstrated a materials–geometry regime on soft, protein‐mimetic hydrogels in which sequence‐defined ELRs and micro/nanogratings act in concert to substantially accelerate iPSC‐EC capture, alignment, and monolayer formation. The novelty of our approach lies not in topography or ELR chemistry alone, but in their integration into a soft, optically clear and rapidly imprintable hydrogel system that links immediate endothelial capture, contact‐guided alignment, and junctional monolayer maturation within the same material platform. This distinguishes the work from prior studies performed mainly on rigid or PDMS substrates, and from ELR hydrogel studies lacking defined micro/nanotopographical control [4, 5, 6, 7, 28].

Our stringent 15‐min attachment assay revealed that ELR chemistry provides a critical baseline for cell adhesion, with all ELR coatings significantly outperforming gelatin. This chemistry‐driven advantage was further modulated by topography, as specific groove regimes (notably the submicron N2 and micron‐scale M1 patterns) enhanced early cell retention. This immediate effect is consistent with established contact‐guidance models, where topographic features bias protrusion dynamics and stabilize nascent focal adhesions against fluid shear, a phenomenon previously documented on rigid substrates [3, 5, 6] and recently extended to soft hydrogels showing anisotropic traction responses [7, 9, 10]. Crucially, these early advantages were not transient; they propagated to the tissue scale. ELR2 and ELR3 on M1/M2 grooves supported the development of confluent, aligned monolayers by day 14, while gelatin remained sparsely populated under identical conditions. Although ELR3 contains the highest density of RGD motifs, the protease‐sensitive ELR2 variant achieved superior endothelial coverage, likely due to a favorable balance between adhesion strength, matrix remodeling, and topographic fidelity [6, 12, 13, 14, 15, 17, 18]. This demonstrates that the initial biophysical cue is locked in through subsequent proliferation and junction maturation, a process enhanced by groove geometries known to polarize the cytoskeleton and direct collective cell migration [4, 5, 6, 10, 28], consistent with junctional remodeling driven by coordinated actomyosin forces along groove orientation [19].

The superior performance of ELR composites is underpinned by their mechanical and structural properties. Rheology confirmed them as elastic‐dominant, shear‐thinning networks, capable of storing elastic energy while permitting local remodeling under cell traction. Cryo‐SEM revealed a fibrillar, interconnected architecture, and tensile testing delineated the mechanical contributions of each ELR: while ELR3 achieved the highest ultimate strength, ELR2 provided the most consistent and highest small‐strain stiffness. This reproducible mechanical integrity, particularly of ELR2, likely resists microtearing during critical phases of colony expansion and merger, preventing the adhesion resets that plague weaker gelatin matrices [4, 28], in agreement with prior work on sequence‐controlled ELRs where tailored stiffness preserves endothelial integrity under cyclic strain [12, 13, 14, 15, 29]. The observed cellular responses likely arise from a combination of mechanisms, including integrin‐mediated adhesion to ELR sequences, contact guidance along microtopographical features, and matrix‐dependent effects such as viscoelasticity and local mechanical cues. Given the well‐established sensitivity of endothelial cells to substrate stiffness and viscoelasticity, the observed differences in adhesion and monolayer formation may partly reflect variations in matrix mechanics in addition to biochemical composition.

Furthermore, we found that chemistry gates the geometric response. NMR confirmed successful ELR incorporation into the composites and allowed us to discriminate between formulations. Functionally, the RGD motifs in ELR3 promote robust initial integrin engagement, whereas the protease‐responsive DRIR sequence in ELR2 appears to slow interfacial remodeling, preserving both ligand availability and topographic fidelity during the critical seeding window. Notably, the RGD‐bearing ELR3 did not consistently outperform the uPA‐responsive ELR2, despite providing a defined integrin‐binding motif. This is likely because gelatin is inherently cell‐adhesive and already supplies multiple integrin‐recognition sequences, making the additional RGD partially redundant in this composite context. In contrast, ELR2 introduces a genuinely new functionality, uPA‐mediated proteolytic remodeling, that is not present in gelatin, which may explain its superior performance in supporting endothelial spreading and monolayer formation on selected patterns. Overall, the consistent outperformance of ELR2 on key patterns suggests that controlling the degradation timeline can be as important as maximizing initial adhesion density for ensuring durable alignment and rapid coverage [2, 3, 5, 6, 10].

From a practical standpoint, the identified ELR2/ELR3 × M1/M2 combinations (microsize grooves of 1 µm) define a readily deployable design window. These pairs are favorable for creating expansion surfaces that minimize preculture handling, optically clear organ‐on‐chip inserts, and prealigned luminal liners for vascular grafts—addressing a long‐standing gap dominated by opaque, rigid materials [1, 13]. Conceptually, our work demonstrates that temporal coupling from seconds to weeks can be engineered by pairing sequence‐encoded matrix properties with feature sizes that match cellular sensing scales [4, 5, 6, 28], linking nanoscale integrin clustering (∼100–350 nm) to multicellular alignment and junction co‐orientation on micron‐scale patterns [8, 9, 19, 21, 22].

### 5.1 Limitations and Future Outlook

All biological experiments in the present study were performed under static culture conditions in order to isolate the effects of matrix bioactivity and surface topography on endothelial attachment and organization. It is well established that endothelial maturation and barrier function are strongly influenced by physiological shear stress. Future studies will therefore investigate the performance of patterned ELR hydrogels under dynamic flow conditions and in curved geometries relevant to vascular constructs. Future studies will evaluate whether patterned ELR matrices remain mechanically stable under perfusion and support aligned endothelial monolayers in tubular geometries. These studies are currently being expanded toward perfusable systems and tubular geometries. Under physiological conditions, shear stress is expected to further influence endothelial alignment, maturation, and barrier function. Although this study was performed under static conditions, future work will focus on evaluating the performance of these materials under controlled flow environments.

This study, however using a single iPSC‐EC line under static conditions, provides a foundational map. The logical next steps involve introducing physiological complexity testing barrier function (TEER, VE‐cadherin continuity), nitric oxide release, and adhesion stability under pulsatile flow, especially in scenarios where flow direction is aligned or misaligned with topographical guidance [5, 6], and examining how flow‐induced stress couples with substrate anisotropy to regulate VE‐cadherin mechanotransduction [7, 19]. Pattern fidelity, particularly for the smallest nanogrooves, could be further optimized with colder imprinting temperatures. Although a reduction in nanoscale feature height was observed after transfer, the remaining topographical features are expected to remain within the relevant scale for integrin clustering and cell–material interactions, although their effect may be less pronounced compared to microscale geometries. Finally, validating this platform across higher aspect ratio designs (perhaps beneficial for cell retention) and multiple iPSC‐EC lines will be essential to demonstrate its generality [16]. This workflow is also compatible with gently curved geometries, such as tubular molds, but these demonstrations remain to be validated experimentally in future work.

## 6 Conclusions

This study introduces patterned ELR–gelatin composite hydrogels as a versatile platform for engineering endothelial interfaces. By integrating programmable recombinamer bioactivity with micro‐ and nanoscale surface architectures, the system enables rapid endothelial cell capture, guided cytoskeletal alignment, and accelerated monolayer formation. The results highlight how matrix bioactivity, mechanics, and topography can be combined to regulate endothelial organization on soft hydrogel substrates. This materials‐design strategy provides a foundation for future development of vascular biomaterials and microphysiological systems requiring controlled endothelialization.

## Author Contributions

J.L. and A.R., conceptualized and designed the experiments. Y.R., M.M., K.T., P.P., D.U., and I.P. were involved in the methodology. J.L., J.C.R.‐C., and A.R. provided resources. J.L. wrote the original draft. All authors were involved in writing, review and editing. J.L. performed statistical analysis. J.L. and A.R. acquired funding and supervised the project. All authors agreed to the authorship.

## Conflicts of Interest

The authors declare no conflicts of interest.

## Supporting information




**Supporting File**: adhm71570‐sup‐0001‐SuppMat.docx.

## Data Availability

All data supporting the findings of this study are presented within the manuscript and its Supporting Information. Any additional information or inquiries can be directed to the corresponding authors upon reasonable request.
