# Supplementary material for: Patterned ELR‐Gelatin Hydrogels Enable Rapid Endothelial Monolayer Formation via Bioactive Matrix Chemistry and Surface Topography
Source: Adv Healthc Mater. 2026 Aug 10;15(34):e71570. doi: 10.1002/adhm.71570 (PMC13568830; doi:10.1002/adhm.71570)
Supplement: Supplementary file 1 [file ADHM-15-0-s001.docx]

**Supplementary Table S1 –** Mapping between full pattern identifiers and abbreviated codes

| **Abbreviation** | **Full name** | **Groove width** | **Height** |
| --- | --- | --- | --- |
| F | Flat | — | — |
| N1 | N350H1 | 350 nm | 1 µm |
| N2 | N350H0.5 | 350 nm | 0.5 µm |
| M1 | M4H1 | 4 µm | 1 µm |
| M2 | M8H1 | 8 µm | 1 µm |
| M3 | M4H0.5 | 4 µm | 0.5 µm |
| M4 | M8H0.5 | 8 µm | 0.5 µm |

**Supplementary Table S2**. List of antibodies and primers.

| **Antibody** | **Type/Use** | **Host** | **Dilution** | **Manufacturer** |
| --- | --- | --- | --- | --- |
| VE-cadherin(IgG) | Primary/IF | Rabbit | 1:200 | Invitrogen, PA5-19612 |
| Phalloidin, Texas Red® | -/IF | - | 1:40 | Life technologies, T7471 |
| Donkey anti-rabbit IgG | Secondary/IF | Donkey | 1:200 | Jackson, 711-545-152  711-605-152 |
| **Primer** | **Sense** | | **Antisense** | |
| ***PECAM1***  ***CDH5*** | AGTCGGACAGTGGGACGTAT  GATTTGGAACCAGATGCACA | | ATGACCTCAAACTGGGCATC  ACTTGGCATTCTTGCGACTC | |
| ***GAPDH*** | GCACCGTCAAGGCTGAGAAC GCACCGTCAAGGCTGAGAAC | | AGGGATCTCGCTCCTGGAA | |

Figure S1 - Surface topography of hydrogel replicas imprinted with line gratings M8H1. Representative bright-field micrographs of ELR1, ELR2, ELR3, gelatin (G), and the PUA master mold, alongside 3D height maps and reconstructions acquired with a digital microscope (VHX-2000, Keyence). Imprints reveal faithful transfer of microscale patterns across different hydrogel formulations.

Figure S2 - Surface topography of hydrogel replicas imprinted with line gratings M8H0.5. Representative bright-field micrographs of ELR1, ELR2, ELR3, gelatin (G), and the PUA master mold, alongside 3D height maps and reconstructions acquired with a digital microscope (VHX-2000, Keyence). Imprints reveal faithful transfer of microscale patterns across different hydrogel formulations.

Figure S3 - Surface topography of hydrogel replicas imprinted with line gratings M4H1. Representative bright-field micrographs of ELR1, ELR2, ELR3, gelatin (G), and the PUA master mold, alongside 3D height maps and reconstructions acquired with a digital microscope (VHX-2000, Keyence). Imprints reveal faithful transfer of microscale patterns across different hydrogel formulations.

Figure S4 - Surface topography of hydrogel replicas imprinted with line gratings M4H0.5. Representative bright-field micrographs of ELR1, ELR2, ELR3, gelatin (G), and the PUA master mold, alongside 3D height maps and reconstructions acquired with a digital microscope (VHX-2000, Keyence). Imprints reveal faithful transfer of microscale patterns across different hydrogel formulations.

Figure S5 - Surface topography of hydrogel replicas imprinted with line gratings N350H1. Representative bright-field micrographs of ELR1, ELR2, ELR3, gelatin (G), and the PUA master mold, alongside 3D height maps and reconstructions acquired with a digital microscope (VHX-2000, Keyence). Imprints reveal faithful transfer of microscale patterns across different hydrogel formulations.

Figure S6 - Surface topography of hydrogel replicas imprinted with line gratings N350H0.5. Representative bright-field micrographs of ELR1, ELR2, ELR3, gelatin (G), and the PUA master mold, alongside 3D height maps and reconstructions acquired with a digital microscope (VHX-2000, Keyence). Imprints reveal faithful transfer of microscale patterns across different hydrogel formulations.

Figure S7 - Mechanical and molecular characterization of gelatin–ELR hydrogels.

(A) True stress–strain curves from individual samples obtained by uniaxial tension testing (Zwick Z100, 60 mm min⁻¹).

(B) Averaged true stress–strain curves.

(C) Box plot summarizing tensile strain at break.

(D) Box plot summarizing Young’s modulus.

(E) Box plot summarizing ultimate tensile strength.

(F) Box plot summarizing stiffness.

(G) Representative images of ELR2 sample during tensile testing.

Figure S8 - Circular histograms showing the orientation distribution of iPSC-derived endothelial cells (iPSC-ECs) cultured under the indicated conditions on gelatin control. Each plot represents the frequency of cell alignment angles relative to the pattern direction.

Figure S9 - Circular histograms showing the orientation distribution of iPSC-derived endothelial cells (iPSC-ECs) cultured under the indicated conditions on ELR1 hydrogels. Each plot represents the frequency of cell alignment angles relative to the pattern direction.

Figure S10 - Circular histograms showing the orientation distribution of iPSC-derived endothelial cells (iPSC-ECs) cultured under the indicated conditions on ELR2 hydrogels. Each plot represents the frequency of cell alignment angles relative to the pattern direction.

Figure S11 - Circular histograms showing the orientation distribution of iPSC-derived endothelial cells (iPSC-ECs) cultured under the indicated conditions on ELR3 hydrogels. Each plot represents the frequency of cell alignment angles relative to the pattern direction.

Figure S12 - Representative low-magnification VE-cadherin immunostaining images of iPSC-derived endothelial cells and HUVEC positive controls. Images provide an overview of endothelial junction formation and monolayer organization complementary to the high-magnification VE-cadherin/F-actin staining shown in Figure 3. VE-cadherin, green; nuclei, DAPI/blue. Scale bars as indicated.
